# Preoperative Evaluation of the Histological Grade of Hepatocellular Carcinoma with Diffusion-Weighted Imaging: A Meta-Analysis

**DOI:** 10.1371/journal.pone.0117661

**Published:** 2015-02-06

**Authors:** Jie Chen, Mingpeng Wu, Rongbo Liu, Siyi Li, Ronghui Gao, Bin Song

**Affiliations:** Department of Radiology, West China Hospital, Sichuan University, Chengdu, Sichuan Province, P. R. China; Affiliated Hospital of North Sichuan Medical College, CHINA

## Abstract

**Objective:**

To evaluate the diagnostic performance of diffusion-weighted imaging (DWI) in the preoperative prediction of the histological grade of hepatocellular carcinoma (HCC).

**Materials and Methods:**

A comprehensive literature search was performed in several authoritative databases to identify relevant articles. QUADAS-2 was used to assess the quality of included studies. Data were extracted to calculate the pooled sensitivity, specificity, positive likelihood ratio (PLR) and negative likelihood ratio (NLR). Summary receiver operating characteristic (SROC) curves were derived and areas under the SROC curve (AUC) were computed to indicate the diagnostic accuracy. Heterogeneity test, meta-regression analysis and sensitivity analysis were performed to identify factors and studies contributed to the heterogeneity.

**Results:**

A total of 11 studies with 912 HCCs were included in this meta-analysis. The pooled sensitivity, specificity, PLR and NLR with corresponding 95% confidence intervals (CI) were 0.54(0.47–0.61), 0.90(0.87–0.93), 4.88(2.99–7.97) and 0.46(0.27–0.77) for the prediction of well-differentiated HCC (w-HCC), 0.84(0.78–0.89), 0.48(0.43–0.52), 2.29(1.43–3.69) and 0.30(0.22–0.41) for the prediction of poorly-differentiated HCC (p-HCC). The AUC were 0.9311 and 0.8513 in predicting w-HCC and p-HCC, respectively. Results were further evaluated according to the method of image interpretation. Significant heterogeneity was observed.

**Conclusion:**

DWI had excellent and moderately high diagnostic accuracy for the detection of w-HCC and p-HCC, respectively. Nonetheless, further studies in larger populations and an optimized image acquisition and interpretation are required before DWI-derived parameters can be used as a useful image biomarker for the prediction of the histological grade of HCC.

## Introduction

Hepatocellular carcinoma (HCC) is a commonly diagnosed malignancy worldwide, especially in the setting of chronic liver disease [1]. Patient management has significantly improved during the last few years [2]. However, the poor prognosis of patients with high grades HCCs after curative treatment remains a challenge [3], with a reported 5-year recurrence rate of up to 70% for liver resection and 15–30% for transplantation [4,5].

The histological grade of HCC is a significant prognostic factor after surgery. High grade HCC was found to be an independent predictor of microvascular invasion (MVI) [6], and was associated with various differentially regulated metastasis and invasion related genes [7,8]. Studies have reported that poorly differentiated HCCs (p-HCC) are associated with worse survival compared with well and moderately differentiated HCC [9,10]. For this reason, it has been suggested by some surgeons that tumor differentiation should be taken into account as well as others factors when the surgical plan was decided [11]. Non-surgical candidates could also benefit from it [6].

Preoperative prediction of the histological grade of HCC is therefore pivotal to treatment planning and prognostication [12,13]. However, the histological grade of HCC is not routinely given by biopsy before surgery for the invasiveness and concern for procedure-related complications [10]. Therefore, many efforts have focused on the non-invasive evaluation of HCC differentiation grade using radiological findings. Recent technique advances in magnetic resonance imaging (MRI) have enabled the evaluation of tumor histological grade through tumor vascularity by dynamic contrast enhanced MRI (DCE-MRI) [14–16], tumor cellularity by diffusion weighted imaging (DWI) [14,17–27] and the hepatocyte function by hepatocyte-specific agents [28–31]. Among them, DWI showed the most promising application. Generally, HCCs with lower apparent diffusion coefficients (ADC) and higher signal intensity on DWI tends to have worse histological grades. Lately, an increasing number of studies have investigated the relationship between the histological grade of HCC and DWI, although discrepant results were reported. In some studies, poorly differentiated HCC (p-HCC) showed significantly lower ADC than well differentiated HCC (w-HCC) and moderately differentiated HCC (m-HCC) [17,18,21], but other studies found poor correlation between ADC and the histological grade of HCC [20,25]. Therefore, this study aims to evaluate the value of DWI in predicting the histological grade of HCC through a synthesis of published experimental researches.

## Materials and Methods

This meta-analysis was carried out in accordance with the recommended PRISMA (Preferred Reporting Items for Systematic Reviews and Meta-Analyses) checklist [32].

### Literature Search and Screening

A comprehensive literature search was performed independently by two investigators in PubMed, EMBASE, Web of science, EBSCO, and The Cochrane Library to identify relevant studies that addressed the diagnostic accuracy of DWI in estimating the histopathological grades of HCC published between January 2004 and July 2014. The search strategy was (((((((carcinoma, hepatocellular) OR hepatocellular carcinoma) OR liver cell carcinoma) OR hepatoma) OR liver cancer)) AND ((((((diffusion magnetic resonance imaging) OR diffusion MRI) OR diffusion weighted imaging) OR DWI) OR apparent diffusion coefficient) OR ADC)) AND (((((histologic* grade) OR histopathologic* grade) OR tumor grading) OR tumor differentiation) OR neoplasm grading). We did not limit our research to publications of certain language.

Inclusion criteria for meta-analysis were (a) DWI was performed in patients with HCC before treatment; (b) patients had pathological proof of the histological grade of HCC; (c) the diagnostic results of true-positive (TP), false-positive (FP), false-negative (FN) and true-negative (TN) were available; (d) the study included at least 20 HCCs. Non-original researches and republished studies were excluded.

### Data Extraction and Quality Assessment

Two investigators independently extracted the following information decided beforehand: author, year of publication, study design (prospectively or retrospectively), patient age, gender (male-to-female ratio), etiology of underlying liver disease, liver function (Child-Pugh A/B/C), number of HCC, mean tumor size, histological differentiation (well/ moderately/ poorly-differentiated), reference standard (liver biopsy and/or surgery), blinding procedure, time intervals between reference standard and index test, imaging protocols adopted to perform DWI (magnetic field strength, b values, image interpretation and diagnostic threshold) and the diagnostic results (TP, FP, FN and TN). Disagreements were resolved by consensus.

Quality assessment of studies eligible for meta-analysis was conducted according to QUADAS-2 (Quality Assessment of Diagnostic Accuracy Studies) [33]. Data extraction and quality assessment were carried out by two investigators on consensus.

### Statistical Analysis and Data Synthesis

The primary outcome was the identification of w-HCC from higher grades and/or p-HCC from lower grades. Results were presented as TP, FP, FN and TN.

The heterogeneity of included results was assessed statistically using the Q statistic of the Chi-square value test and the inconsistency index (I^2^). The I^2^ index is a measure of the percentage of the total variation across studies resulting from heterogeneity beyond chance. I^2^＞50% or p＜0.1 for the Chi-square value test indicate the presence of significant heterogeneity [34]. If significant heterogeneity was observed, a random-effects coefficient binary regression model was used to summarize the pooled diagnostic performance accordingly [35]. The summary receiver operating characteristic (SROC) curve was constructed and areas under the SROC curve (AUC) were consulted when determining the diagnostic performance [36]. The prediction of w-HCC and p-HCC were evaluated separately, and results were further evaluated according to the image interpretation.

Threshold effect was judged visually and quantitatively through the SROC curve and Spearman correlation coefficient between the logit of sensitivity and the logit of (1－specificity). A strong positive correlation with p ＜ 0.05 would suggest the existence of threshold effect [37]. Meta-regression analysis was performed on study design, age, male-to-female ratio, number of HCC, tumor size, reference standard, blinding procedure, and imaging protocols to further explore variables contributed to the heterogeneity if necessary. In addition, sensitivity analysis was performed to ensure the reliability of included studies and to identify potential studies that may cause notable heterogeneity. Data analyses were performed using the Meta-DiSc software (version 1.4) [38].

The potential publication bias was assessed by the Deek’s funnel plot and an asymmetry test using Stata software (version 12.0). An asymmetrical funnel plot with p ＜ 0.05 would indicate the presence of publication bias [39].

## Results

### Literature Search

**Fig 1:**
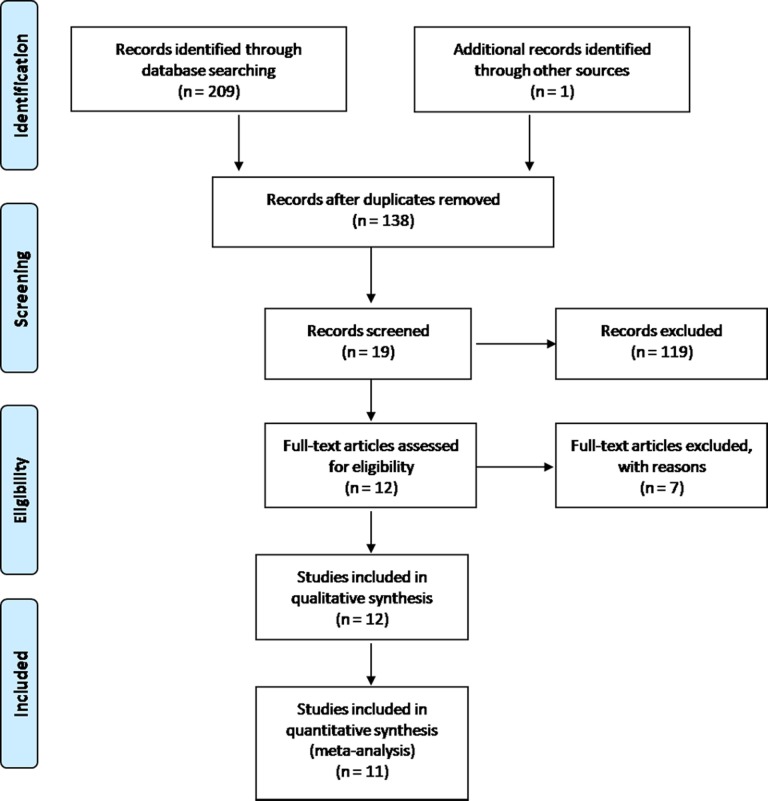
Flow diagram of study selection.

The systematic search initially yielded 210 results. 138 records were identified after duplicates removed. 119 studies were excluded following title and abstract screening. After reading the full texts, 7 of the remaining 19 articles were excluded. Finally, 12 studies [14,17–27] were included for qualitative analysis, of which 11 studies were eligible for meta-analysis. The study selection process is presented with a flowchart in Fig. 1.

### Study Characteristics and Quality Assessment

In the 11 studies included for meta-analysis, a total of 912 HCCs were included, with 239 w-HCCs, 449 m-HCCs and 224 p-HCCs. All studies were designed retrospectively. Blinding procedure was reported in 8 studies. Patients were included consecutively in most studies with or without the history of underlying liver disease (hepatitis B/C infection, fibrosis, cirrhosis and steatohepatitis, etc.). Liver function was reported only in 4 of the 11 studies. Two studies that included only hypervascular HCCs were specifically noticed. In studies [14,18] where the histopathological grades were presented as the Edmondson-Steiner grade, tumor differentiation was classified into well, moderate and poor according to Edmondson-Steiner’s grading system [40].

There were 2 studies had outcome data for the prediction of w-HCC, 4 studies had outcome data for the prediction of p-HCC, and 5 studies had outcome data for the prediction of both w-HCC and p-HCC. DWI was interpreted qualitatively by visual assessment (VA) and quantitatively by ADC quantification (ADC-Q) in 4 and 2 studies respectively in predicting w-HCC, with one study reported the outcome data of both qualitative and quantitative interpretation. For the prediction of p-HCC, DWI was interpreted qualitatively and quantitatively in 4 and 5 studies, respectively. Therefore, a total of 7 studies with 8 data sets were available for the prediction of w-HCC, and 9 studies with 9 data sets were available for the prediction of p-HCC.

**Fig 2:**
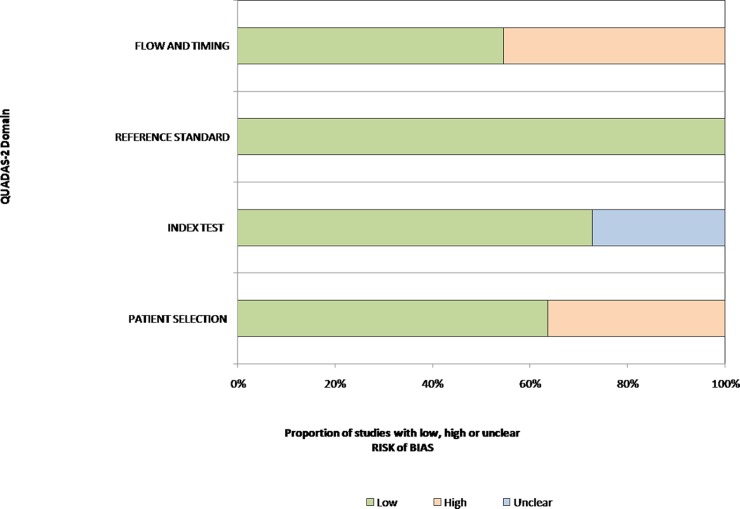
Graphical display for QUADAS-2 results regarding proportion of studies with high, low or unclear risk level of bias. Results showed that a considerable risk of bias existed in flow and timing, which was mainly caused by different reference standard and unclear time intervals between index test and reference standard.

Principal information about those included studies was summarized in Table 1. The included studies had high quality. Result of quality assessment for the 11 studies was presented in Table 2. Fig. 2 shows a graphical display for QUADAS-2 results regarding the proportion of studies with low, high or unclear risk of bias.

**Table 1:** Characteristics of the 11 studies included in the meta-analysis.

Author	Nation	Year	FS	B value	De	Blind	RS	TI	No. HCCs	Age	Size	TH	TP	FP	FN	TN	W/P	Inter
Chang et al	Taiwan	2014	1.5	0,500	retro	Yes	S	9.4	141	61.9	3.73	1.7	28	18	6	89	W	ADC-Q
	Taiwan	2014	1.5	0,500	retro	Yes	S	9.4	141	61.9	3.73	1.4	36	10	7	88	P	ADC-Q
Woo et al	Korea	2013	3	0,25,50,75,100,200,500,800	retro	Yes	S	17	42	55.5	4.7	1.14	13	6	5	18	P	ADC-Q
Sandrasegaran et al	USA	2012	1.5	0,50,400,500, 800	retro	Yes	B	?	41	55	4.4	0.99	12	4	2	23	W	ADC-Q
	USA	2012	1.5	0, 50	retro	Yes	B	?	41	55	4.4	—	5	4	9	23	W	VA
An et al	Korea	2012	3	0,50,400,800	retro	Yes	S	?	201	57	2.7	—	23	8	14	156	W	VA
	Korea	2012	3	0, 50, 400, 800	retro	Yes	S	?	201	57	2.7	—	33	137	1	30	P	VA
Saito et al	Japan	2012	1.5	100,800	retro	Yes	B	?	42	69	1.83	—	13	2	4	23	W	VA
	Japan	2012	1.5	100, 800	retro	Yes	B	?	42	69	1.83	—	7	20	0	15	P	VA
Nakanishi et al	Japan	2012	1.5	50,1000	retro	Unclear	S	＜30	50	61	5.7	0.8	14	6	4	26	P	ADC-Q
Nishie et al	Japan	2011	1.5	0,500,1000	retro	Unclear	S	＜30	85	65	3.6	0.972	19	16	7	43	P	ADC-Q
Heo et al	Korea	2010	1.5	0,1000	retro	Yes	S	11	27	57	5.6	0.99	7	3	2	15	P	ADC-Q
Muhi et al	Japan	2009	1.5	500,1000	retro	Yes	B	8	86	62.7	2.4	0.92	25	4	14	43	W	ADC-Q
Nasu et al	Japan	2009	1.5	0,500	retro	Unclear	S	18.3	125	64.8	2.9	—	3	8	22	92	W	VA
	Japan	2009	1.5	0,500	retro	Unclear	S	18.3	125	64.8	2.9	—	33	54	6	32	P	VA
Piana et al	France	2011	1.5	0, 500	retro	Yes	B	45	72	63	2.2	—	7	4	28	33	W	VA
	France	2011	1.5	0, 500	retro	Yes	B	45	72	63	2.2	—	8	53	1	10	P	VA

FS, field strength (Tesla); De, design; RS, reference standard (S, surgical specimens; B, biopsy or surgical specimens); TI, time intervals between DWI and reference standard (days); TH, the diagnostic threshold of ADC (×10^﹣3^mm^2^/s); TP, true positive; FP, false positive; FN, false negative; TN, true negative; W, the prediction of well-differentiated HCC; P, the prediction of poorly-differentiated HCC; Inter, image interpretation; ADC-Q, ADC quantification; VA, visual assessment.

**Table 2:** Quality assessment of the 11 included diagnostic studies.

Study	RISK OF BIAS				APPLICABILITY CONCERNS		
	PATIENT SELECTION	INDEX TEST	REFERENCE STANDARD	FLOW AND TIMING	PATIENT SELECTION	INDEX TEST	REFERENCE STANDARD
Chang et al	☺	☺	☺	☺	☺	☺	☺
Woo et al	☺	☺	☺	☺	☺	☺	☺
Sandrasegaran et al	☹	☺	☺	☹	☺	☺	☺
An et al	☺	☺	☺	☹	☺	☺	☺
Saito et al	☺	☺	☺	☹	☺	☺	☺
Nakanishi et al	☺	?	☺	☺	☺	☺	☺
Nishie et al	☺	?	☺	☺	☺	☺	☺
Heo et al	☹	☺	☺	☺	☺	☺	☺
Muhi et al	☺	☺	☺	☹	☺	☺	☺
Nasu et al	☹	?	☺	☺	☺	☺	☺
Piana et al	☹	☺	☺	☹	☺	☺	☺

^☺^Low Risk ^☹^High Risk ? Unclear Risk

### Diagnostic Accuracy for the Prediction of Well-differentiated HCC

**Fig 3:**
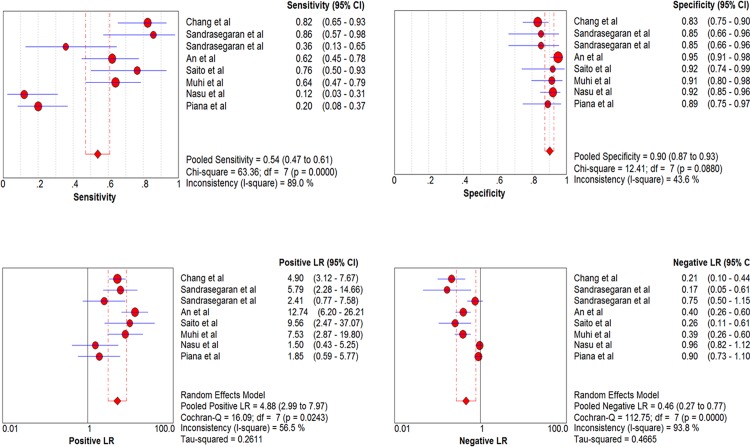
Forest plots of the estimates for DWI in predicting w-HCC. The Q statistics and I^2^ indexes of specificity suggested the presence of notable heterogeneity, and the diagnostic performance was summarized by using a random-effects coefficient binary regression model.

The overall pooled sensitivity and specificity with corresponding 95% confidence intervals (95% CI) in predicting w-HCC were 0.54 (0.47–0.61) and 0.90 (0.87–0.93), respectively. Sensitivity of individual studies ranged widely from 12% to 86%, while specificity focused mainly from 83% to 95%. The pooled positive likelihood ratio (PLR) and negative likelihood ratio (NLR) were 4.88 (2.99–7.97) and 0.46 (0.27–0.77), respectively. According to the SROC curve, the AUC was 0.9311. There was significant heterogeneity (I^2^ = 89%) in the sensitivity between each study. No threshold effect was detected. Meta-regression analysis revealed no factors contributed significantly to the heterogeneity. Forest plots of sensitivity, specificity, PLR and NLR are shown in Fig. 3.

Subgroup analysis was performed according to the method of image interpretation. VA yielded a lower sensitivity (40%) and higher specificity (93%) than that of ADC-Q (75% and 86%, respectively). Notable heterogeneity existed in the pooled sensitivity for VA (I^2^ = 87.8%), while only slightly significant heterogeneity (I^2^ = 53.5%) existed in the pooled sensitivity for ADC-Q.

Two studies that included only hypervascular HCCs and showed extremely low sensitivity were excluded for sensitivity analysis. Therefore, when combined, the pooled sensitivity, specificity, PLR and NLR for the remaining studies were 0.68 (0.60–0.76), 0.90 (0.87–0.93), 6.35 (4.08–9.88) and 0.36 (0.23–0.57) for overall evaluation, and 0.60 (0.48–0.72), 0.94 (0.89–0.96), 6.96 (2.45–19.76) and 0.46 (0.24–0.85) for VA. Along with the significant decrease of heterogeneity, the pooled sensitivity increased considerably in both groups. The diagnostic results of subgroup analysis and sensitivity analysis were presented in Table 3.

**Table 3:** Results of subgroup analysis and sensitivity analysis.

Study	No	Sensitivity	I^2^	Specificity	I^2^	PLR	NLR	AUC
***Well differentiated HCC***								
**Overall**	*8*	0.54(0.47–0.61)	89%	0.90(0.87–0.93)	43.6%	4.88(2.99–7.97)	0.46(0.27–0.77)	0.9311
**Subgroup analysis**								
VA	*5*	0.40(0.31–0.49)	87.8%	0.93(0.89–0.95)	1.2%	3.98(1.53–10.35)	0.63(0.40–1.00)	0.9705
ADC-Q	*3*	0.75(0.64–0.83)	53.5%	0.86(0.80–0.90)	0.3%	5.36(3.69–7.79)	0.28(0.16–0.50)	0.9035
**Sensitivity analysis**								
Overall	*6*	0.68(0.60–0.76)	62.8%	0.90(0.87–0.93)	58.1%	6.35(4.08–9.88)	0.36(0.23–0.57)	0.9101
VA	*3*	0.60(0.48–0.72)	63.5%	0.94(0.89–0.96)	36.8%	6.96(2.45–19.76)	0.46(0.24–0.85)	0.9618
***Poorly differentiated HCC***								
**Overall**	*9*	0.84(0.78–0.89)	38.7%	0.48(0.43–0.52)	96.4%	2.29(1.43–3.69)	0.30(0.22–0.41)	0.8513
**Subgroup analysis**								
VA	*4*	0.91(0.83–0.96)	40.2%	0.25(0.20–0.30)	84.5%	1.25(1.08–1.45)	0.37(0.19–0.72)	0.6316
ADC-Q	*5*	0.78(0.69–0.85)	0.0%	0.82(0.77–0.87)	52.2%	4.10(2.55–6.58)	0.28(0.20–0.40)	0.7822
**Sensitivity analysis**								
Overall	*7*	0.83(0.76–0.89)	53.2%	0.54(0.49–0.59)	96.7%	2.98(1.15–7.70)	0.28(0.20–0.39)	0.8630
VA	*2*	0.98(0.87–1.00)	0.0%	0.22(0.17–0.29)	89.1%	1.35(0.96–1.88)	0.16(0.03–0.77)	/

VA, visual assessment; ADC-Q, ADC quantification; No, number of data sets; I^2^, corresponding inconsistency index (I^2^) of the pooled sensitivity and specificity; PLR, positive likelihood ratio; NLR, negative likelihood ratio; AUC, areas under the SROC curve. Numbers in parentheses are 95% confidence intervals.

### Diagnostic Accuracy for the Prediction of Poorly-differentiated HCC

**Fig 4:**
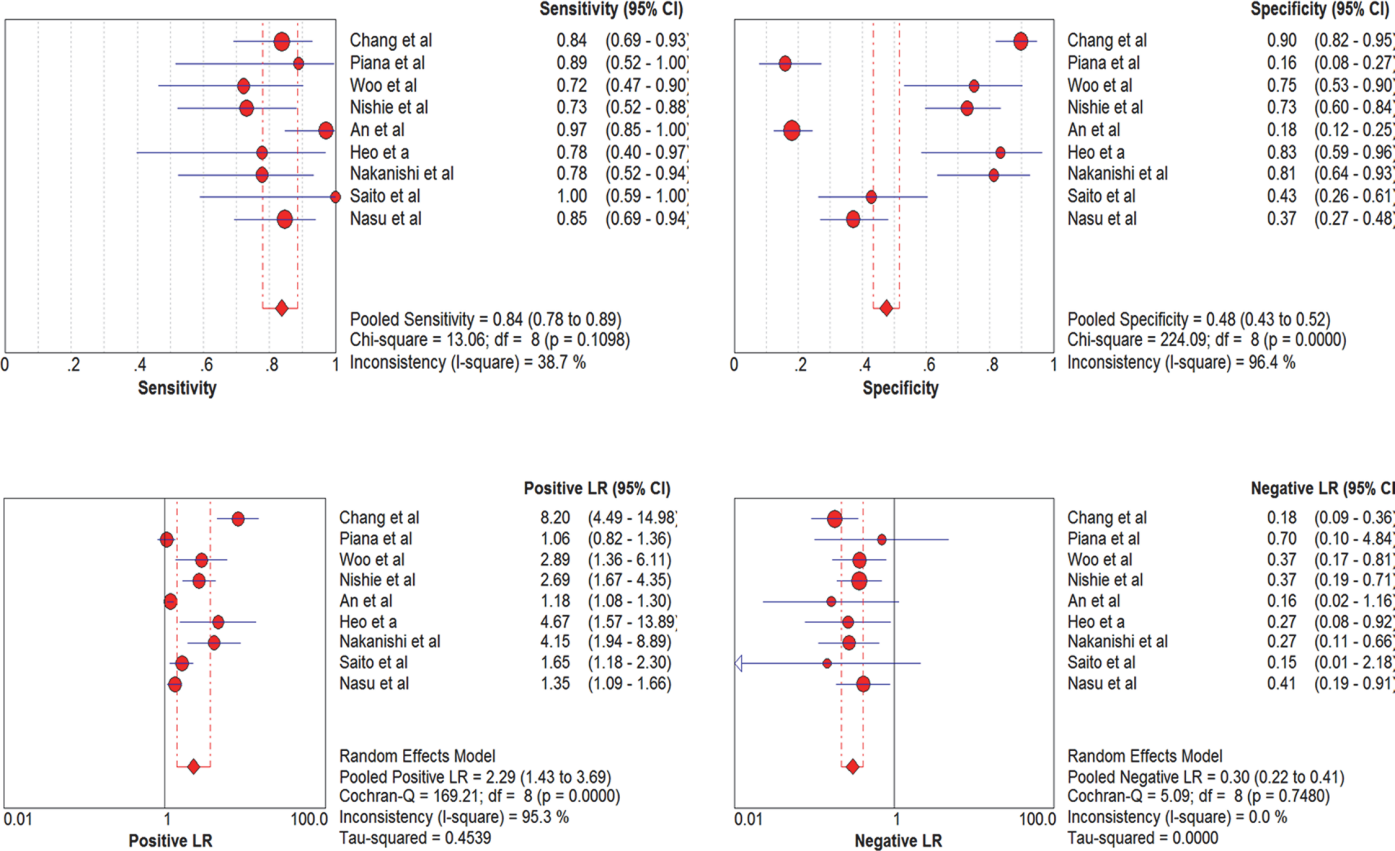
Forest plots of the estimates for DWI in predicting p-HCC. The Q statistics and I^2^ indexes of specificity suggested the presence of notable heterogeneity, and the diagnostic performance was summarized by using a random-effects coefficient binary regression model.

**Fig 5:**
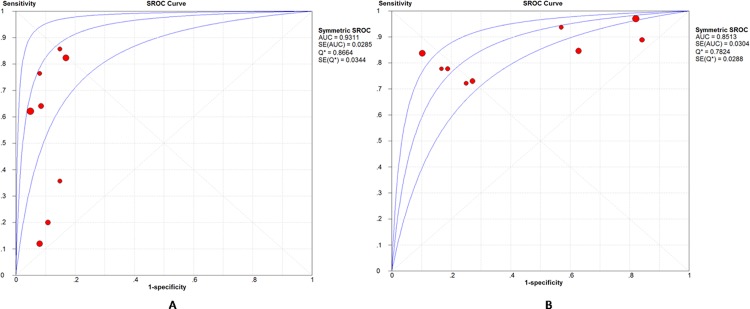
Summary receiver operating characteristic (SROC) curves for DWI in predicting w-HCC and p-HCC. (A) SROC curve for the prediction of w-HCC. (B) SROC curve for the prediction of p-HCC. The AUC was 0.9311 and 0.8513 according to SROC curves, indicating an excellent and moderately high diagnostic accuracy for the prediction of w-HCC and p-HCC, respectively.

There were 9 studies assessed the performance of DWI in predicting p-HCC. Sensitivity of individual studies concentrated within the range of 72% to 100%, while the specificity ranged widely from 16% to 90%. Combined together, the included 9 studies yielded a sensitivity of 0.84 (95% CI, 0.78–0.89) and a relatively low specificity of 0.48 (95% CI, 0.43–0.52). The pooled PLR and NLR were 2.29 (95% CI, 1.43–3.69) and 0.30 (95% CI, 0.22–0.41), respectively. The AUC was 0.8513. Significant heterogeneity was observed in the specificity (I^2^ = 96.4%) between included studies. There was no notable threshold effect in the evaluated 9 studies. Meta-analysis did not found any factors that contributed significantly to the heterogeneity. Forest plots of sensitivity, specificity, PLR and NLR are shown in Fig. 4. SROC curves for the prediction of both w-HCC and p-HCC are shown together in Fig. 5.

Subgroup analysis showed that VA had a high sensitivity of 91% but an extremely low specificity of 25%. Significant heterogeneity still existed (I^2^ = 84.5%) in specificity within the VA group. While ADC-Q had a more agreeable sensitivity of 78% and specificity of 82%, with only slightly significant heterogeneity (I^2^ = 52.2%) in specificity.

For the same reason stated above, two studies were excluded for sensitivity analysis. Results of sensitivity analysis were similar with that of before exclusion for both overall evaluation and VA. The diagnostic results were summarized in Table 3.

### Publish bias

The results of the Deeks’s funnel plot asymmetry test (p = 0.73 and p = o.34) indicated the absence of notable publication bias for the prediction of both w-HCC and p-HCC.

## Discussion

Low grade HCC tends to show hyperintensity on unenhanced T1-weighted imaging and hypointensity on T2-weighted imaging [41]. Morphological evaluation showed that larger tumor size and extrahepatic extension were associated with higher histological grade [17]. However, it is often challenging to determine the differentiation of HCC seen on MR images, especially in patients with cirrhosis because of the architectural distortion of the liver parenchyma and the overlapping imaging appearances [42,43]. Fundamentally different from the conventional morphologic-based imaging techniques, DWI probes the function of tissues. Restricted diffusion of water for a malignant tumor is mainly caused by increased cellular density and decreased extracellular space, and is presented with hyperintensity on DWI or decreased ADC [44]. Earlier study concluded that as histological grade rise, the ADC value tends to decrease, and lesions were more likely to show hyperintensity on DWI [24].

To our knowledge, this is the first meta-analysis of the diagnostic performance of DWI in predicting the histological grade of HCC. Results showed that for differentiating w-HCC from higher grades, DWI had a relatively low sensitivity (54%), high specificity (90%), and an excellent diagnostic performance (AUC = 0.9311). When differentiating p-HCC from lower grades, the pooled sensitivity was 84%, and specificity was 48%, with a moderately high diagnostic performance (AUC = 0.8513). We evaluated the diagnostic performance for the prediction of w-HCC and p-HCC separately because they have significantly different prognosis and ask for different patient management. For instance, when selecting candidates for liver transplantation, consideration may be given to excluding patients with poorly differentiated HCC [12], and include patients with tumors larger than 5 cm with a well differentiated histology [9,45].

There was significant heterogeneity in the pooled sensitivity for the prediction of w-HCC and in the pooled specificity for the prediction of p-HCC. Spearman correlation coefficient confirmed the absence of threshold effect, and meta-regression analysis did not find any factor contributed statistically to the heterogeneity. Sensitivity analysis found that patient inclusion of only hypervascular lesion was the main cause of heterogeneity in the sensitivity for the prediction of w-HCC, but barely affected the diagnostic results for the prediction of p-HCC.

Among the included studies, Nasu et al [25] and Piana et al [26] reported an extremely low sensitivity for predicting w-HCC and an extremely low specificity for predicting p-HCC. We speculate this could be explained by the inclusion of only hypervascular (i.e., arterial enhancement or typical enhancement patterns) HCCs. Studies suggested that the presence of typical enhancement patterns on CT or MRI indicates not only the diagnosis of HCC, but also the potential of MVI, and is more likely to have higher histological grade [15,46].

In 2009, Nasu et al [25] reported that there was no significant correlation between ADC and the histological grade of hypervascular HCC, but slightly significant correlation existed between signal intensity on DWI and histological grade. Following researches included HCCs with histopathological proof of the differentiation grades, regardless of the contrast-enhanced appearances. Heo et al [23] investigated the correlation between the histological grade and both ADC and the expression of vascular endothelial growth factor (VEGF). Results showed that tumor differentiation had a significantly inverse correlation with the ADC value of HCC (r = －0.51), but there was no correlation between the histological differentiation and the VEGF expression (r = －0.33). Another study found the minimum-spot ADC was an independent risk factor for early tumor recurrence after HCC resection, suggesting that DWI has a potential role for histological tumor grading and prediction of early HCC recurrence [21]. Recently, Chang et al [17] reported that ADC value and relative intensity ratio in arterial phase (RIRa) were the two most promising quantitative MRI parameters to distinguish histological grade. Moreover, comparison of the two parameters revealed that the ADC values were more sensitive than RIRa in differentiating w-HCC from higher grades. In summary, all those reports indicated the potential of DWI in predicting the histological grade o f HCC.

In the subgroup analysis, we compared the effect of different image interpretation. Qualitative interpretation was carried out through visually assessing the signal intensity (hypointense, isointense or hyperintense) on DWI images, and hypointensity or isointensity was considered an indicator for w-HCC in this study. An objective VA depends largely on blinding image interpretation, and is affected by the conspicuity of lesion on DWI, especially when lesions were smaller than 1 cm in diameter [47]. Quantitative interpretation, on the other hand, evaluates tumor cellularity through the calculation of ADC. However, the ADC quantification (ADC-Q) can be easily affected by DWI sequence parameters, such as the use of parallel imaging technique and the set of b values [48,49], and the substantial overlap of ADC between different histological grades [22,23] makes it even more trick to estimate tumor differentiation. A previous study suggested that in the setting of solid focal liver lesions, VA was less accurate than ADC-Q for detecting HCC [50]. Results of this study indicated that for the prediction of w-HCC, ADC-Q had the highest sensitivity, while VA demonstrated the highest specificity. When differentiating p-HCC, however, VA demonstrated the best sensitivity, while ADC-Q showed the best specificity. Concerning both the sensitivity and specificity, results of quantitative interpretation (ADC-Q) seem to be more favorable for the prediction of both w-HCC and p-HCC.

There are as yet no standardized imaging protocols and result interpretations regarding the prediction the histological grade of HCC using DWI. In addition to the commonly applied signal intensity on DWI and the average ADC values on ADC maps, few other parameters such as the minimum-spot ADC [21], contrast-to-noise (C/N) ration [20] and relative contrast ratio (RCR) [27] on DWI have been explored. Despite some limitations, so far, most studies preferred the application of ADC [51]. Recent technique advance has promoted the application of intravoxel incoherent motion (IVIM) diffusion-weighted imaging in predicting the histological grade of HCC. By using the intravoxel incoherent motion model and multiple b values, pure diffusion characteristics can be separated from pseudodiffusion caused by perfusion [52]. According to Woo et al [18], IVIM-derived D values (diffusion coefficient, representing pure molecular diffusivity) showed significantly better diagnostic performance than ADC values in differentiating high grade HCC from low grade HCC. It was also proven to have good reproducibility [53]. Hopefully, this could improve the performance of quantitative DWI in predicting the histological grade of HCC. However, considering the limited number of study, further studies with a larger study population are still needed.

Liver function was another concern that may affect the estimation of HCC differentiation. If patients had severely impaired liver parenchyma (fibrosis or cirrhosis), the ADC measurements might show different results [54]. We could not further analyze it because liver function was reported in only few studies. Previous study indicated a tendency toward decreased detection of HCC in DWI with the severity of cirrhosis [42]. Another research reported that the hepatobiliary phase (HBP) of gadoxetic acid-enhanced MRI (EOB-MRI) predicts the histological grade of HCC only in patients with Child-Pugh class A cirrhosis [55], but there is no similar exploration concerning DWI. However, comparison of the pathological results of the background liver parenchyma by Chang et al [17] revealed no significant differences in presence of liver fibrosis, fatty change, or iron deposition between Child-Pugh class A and Child-Pugh B/C patients. This, we speculate, could ease the concern to some degree, but studies are still needed to verify it.

There are still many challenges in the preoperative evaluation of the histological grade of HCC. Generally, it is hard to distinguish well-differentiated HCCs from benign hepatocellular nodules, especially high grade dysplastic nodule [24,56]. Moreover, lesions that are small or located in areas vulnerable to the cardiac motion-related artifacts such as the liver dome and the left subphrenic region cannot be evaluated precisely by visual assessment or by using ADC measurements [57,58]. Although many MRI sequences (conventional T1/T2-weighted [41], DCE-MRI [16,17,59], HBP images of EOB-MRI [29,60] and DWI) have been explored in the prediction of HCC differentiation, no consensus has been obtained yet. So far, there is only one study [14] investigated the utility of combined MRI techniques, and concluded that the combination of DWI and subtraction imaging can help better distinguish the histological grade of HCC. Larger prospective studies should be conducted to further validate the applicability of combined MRI sequences, and to determine the optimal diagnostic algorithm.

Some limitations of this meta-analysis should be addressed. First, the sample size was relatively small. Due to the limited number of studies and information, further analysis was infeasible, and results of this study should also be consulted with caution. Second, the retrospective study design with patients scheduled for surgical resection or transplantation in many studies might have introduced some bias in patient selection, and blinding procedure was unclear in 3 studies, causing concerns about the quality of included studies. Third, although meta-regression analysis found the mean tumor size did not contribute statistically to the heterogeneity, difference did exist in the inclusion of HCC lesions with only three studies included lesions smaller than 1 cm in diameter, which was considered difficult to visualize and to set the region of interest in most studies. Forth, due to the retrospective nature, 3 studies took either surgical or biopsy results as reference standard. However, determining tumor grade on biopsy may be misleading, as tumor grade is often underestimated in core biopsy specimens in comparison with surgical specimens [61,62], and the Interrater disagreement for biopsy evaluation was substantial [61]. Therefore, both the reliability and reproducibility of the grading of HCC using biopsy was queried.

In conclusion, this meta-analysis showed that DWI had excellent and moderately high diagnostic accuracy for the detection of w-HCC and p-HCC, respectively. Although difficulty existed in correct prediction of the histological grade of individual lesion at present, quantitative DWI still holds tremendous potential in the non-invasive prediction of the histological grade of HCC preoperatively. Further studies in larger populations and an optimized image acquisition and interpretation are required before DWI-derived parameters can be used as a useful image biomarker for the prediction of the histological grade of HCC.

## Supporting Information

S1 PRISMA Checklist PRISMA 2009 Checklist. (DOC)

## References

[1] Rampone B, Schiavone B, Confuorto G (2010) Current management of hepatocellular cancer. Curr Oncol Rep 12: 186–192. 10.1007/s11912-010-0094-3 20425078

[2] de Lope CR, Tremosini S, Forner A, Reig M, Bruix J (2012) Management of HCC. J Hepatol 56: 60009–60009. 10.1016/S0168-8278(12)60009-9 22300468

[3] Zhou L, Rui JA, Wang SB, Chen SG, Qu Q (2014) Clinicopathological Predictors of Poor Survival and Recurrence After Curative Resection in Hepatocellular Carcinoma Without Portal Vein Tumor Thrombosis. Pathol Oncol Res 8: 8. 10.1007/s12253-014-9798-2 24908141

[4] Llovet JM, Schwartz M, Mazzaferro V (2005) Resection and liver transplantation for hepatocellular carcinoma. Semin Liver Dis 25: 181–200. 15918147 10.1055/s-2005-871198

[5] Bruix J, Sherman M (2005) Management of hepatocellular carcinoma. Hepatology 42: 1208–1236. 16250051 10.1002/hep.20933

[6] Kim Bk, Han KH, Park YN, Park MS, Kim KS, et al (2008) Prediction of microvascular invasion before curative resection of hepatocellular carcinoma. J Surg Oncol 97: 246–252. 18095300 10.1002/jso.20953

[7] Yu GR, Kim SH, Park SH, Cui XD, Xu DY, et al (2007) Identification of molecular markers for the oncogenic differentiation of hepatocellular carcinoma. Exp Mol Med 39: 641–652. 18059140 10.1038/emm.2007.70

[8] Murakami Y, Tamori A, Itami S, Tanahashi T, Toyoda H, et al (2013) The expression level of miR-18b in hepatocellular carcinoma is associated with the grade of malignancy and prognosis. BMC Cancer 13: 1471–2407. 10.1186/1471-2407-13-99 PMC3600030 23496901

[9] Jonas S, Bechstein WO, Steinmuller T, Herrmann M, Radke C, et al (2001) Vascular invasion and histopathologic grading determine outcome after liver transplantation for hepatocellular carcinoma in cirrhosis. Hepatology 33: 1080–1086. 11343235 10.1053/jhep.2001.23561

[10] Pérez-Saborido B, de los Galanes SJ, Menéu-Díaz JC, Romero CJ, Elola-Olaso AM, et al (2007) Tumor recurrence after liver transplantation for hepatocellular carcinoma: recurrence pathway and prognostic factors. Transplant Proc 39: 2304–2307. 17889172 10.1016/j.transproceed.2007.06.059

[11] Oishi K, Itamoto T, Amano H, Fukuda S, Ohdan H, et al (2007) Clinicopathologic features of poorly differentiated hepatocellular carcinoma. J Surg Oncol 95: 311–316. 17326126 10.1002/jso.20661

[12] Cillo U, Vitale A, Bassanello M, Boccagni P, Brolese A, et al (2004) Liver transplantation for the treatment of moderately or well-differentiated hepatocellular carcinoma. Ann Surg 239: 150–159. 14745321 10.1097/01.sla.0000109146.72827.76 PMC1356206

[13] Suh KS, Cho EH, Lee HW, Shin WY, Yi NJ, et al (2007) Liver transplantation for hepatocellular carcinoma in patients who do not meet the Milan criteria. Dig Dis 25: 329–333. 17960068 10.1159/000106913

[14] An C, Park MS, Jeon HM, Kim YE, Chung WS, et al (2012)—Prediction of the histopathological grade of hepatocellular carcinoma using qualitative diffusion-weighted, dynamic, and hepatobiliary phase MRI. Eur Radiol 22: 1701–1708. 10.1007/s00330-012-2421-6 22434421

[15] Choi YS, Rhee H, Choi JY, Chung YE, Park YN, et al (2013) Histological characteristics of small hepatocellular carcinomas showing atypical enhancement patterns on gadoxetic acid-enhanced MR imaging. J Magn Reson Imaging 37: 1384–1391. 10.1002/jmri.23940 23172629

[16] Choi JW, Lee JM, Kim SJ, Yoon JH, Beak JH, et al (2013) Hepatocellular carcinoma: imaging patterns on gadoxetic acid-enhanced MR Images and their value as an imaging biomarker. Radiology 267: 776–786. 10.1148/radiol.13120775 23401584

[17] Chang WC, Chen RC, Chou CT, Lin CY, Yu CY, et al (2014) Histological grade of hepatocellular carcinoma correlates with arterial enhancement on gadoxetic acid-enhanced and diffusion-weighted MR images. Abdom Imaging 29: 29. 10.1007/s00261-014-0168-z 24869790

[18] Woo S, Lee JM, Yoon JH, Joo l, Han JK, et al (2014) Intravoxel incoherent motion diffusion-weighted MR imaging of hepatocellular carcinoma: correlation with enhancement degree and histologic grade. Radiology 270: 758–767. 10.1148/radiol.13130444 24475811

[19] Sandrasegaran K, Tahir B, Patel A, Ramaswamy R, Bertrand K, et al (2013) The usefulness of diffusion-weighted imaging in the characterization of liver lesions in patients with cirrhosis. Clin Radiol 68: 708–715. 10.1016/j.crad.2012.10.023 23510619

[20] Saito K, Moriyasu F, Sugimoto K, Nishio R, Saguchi T, et al (2012) Histological grade of differentiation of hepatocellular carcinoma: comparison of the efficacy of diffusion-weighted MRI with T2-weighted imaging and angiography-assisted CT. J Med Imaging Radiat Oncol 56: 261–269. 10.1111/j.1754-9485.2012.02374.x 22697322

[21] Nakanishi M, Chuma M, Hige S, Omatsu T, Yokoo H, et al (2012) Relationship between diffusion-weighted magnetic resonance imaging and histological tumor grading of hepatocellular carcinoma. Ann Surg Oncol 19: 1302–1309. 10.1245/s10434-011-2066-8 21927976

[22] Nishie A, Tajima T, Asayama Y, Ishigami K, Kakihara D, et al (2011) Diagnostic performance of apparent diffusion coefficient for predicting histological grade of hepatocellular carcinoma. Eur J Radiol 80: 8. 10.1016/j.ejrad.2010.06.019 20619566

[23] Heo SH, Jeong YY, Shin SS, Kim JW, Lim HS, et al (2010) Apparent diffusion coefficient value of diffusion-weighted imaging for hepatocellular carcinoma: correlation with the histologic differentiation and the expression of vascular endothelial growth factor. Korean J Radiol 11: 295–303. 10.3348/kjr.2010.11.3.295 20461183 PMC2864856

[24] Muhi A, Ichikawa T, Motosugi U, Sano K, Matsuda M, et al (2009) High-b-value diffusion-weighted MR imaging of hepatocellular lesions: estimation of grade of malignancy of hepatocellular carcinoma. J Magn Reson Imaging 30: 1005–1011. 10.1002/jmri.21931 19856432

[25] Nasu K, Kuroki Y, Tsukamoto T, Nakajima H, Mori K, et al (2009) Diffusion-weighted imaging of surgically resected hepatocellular carcinoma: imaging characteristics and relationship among signal intensity, apparent diffusion coefficient, and histopathologic grade. AJR Am J Roentgenol 193: 438–444. 10.2214/AJR.08.1424 19620441

[26] Piana G, Tringuart L, Meskine N, Barrau V, Beers BV, et al (2011) New MR imaging criteria with a diffusion-weighted sequence for the diagnosis of hepatocellular carcinoma in chronic liver diseases. J Hepatol 55: 126–132. 10.1016/j.jhep.2010.10.023 21145857

[27] Le Moigne F, Boussel L, Haguin A, Bancel B, Ducerf C, et al (2014) Grading of small hepatocullular carcinomas (</ = 2cm): correlation between histology, T2 and diffusion-weighted imaging. Br J Radiol 9: 20130763. 10.1259/bjr.20130763 PMC4453134 25007142

[28] Kogita S, Imai Y, Okada M, Kim T, Onishi H, et al (2010) Gd-EOB-DTPA-enhanced magnetic resonance images of hepatocellular carcinoma: correlation with histological grading and portal blood flow. Eur Radiol 20: 2405–2413. 10.1007/s00330-010-1812-9 20490505

[29] Choi JY, Kim MJ, Park YN, Lee JM, Yoo SK, et al (2011) Gadoxetate disodium-enhanced hepatobiliary phase MRI of hepatocellular carcinoma: correlation with histological characteristics. AJR Am J Roentgenol 197: 399–405. 10.2214/AJR.10.5439 21785086

[30] Chou CT, Chen YL, Su WW, Wu HK, Chen RC (2010) Characterization of cirrhotic nodules with gadoxetic acid-enhanced magnetic resonance imaging: the efficacy of hepatocyte-phase imaging. J Magn Reson Imaging 32: 895–902. 10.1002/jmri.22316 20882620

[31] Tahir B, Sandrasegaran K, Ramaswamy R, Bertrand K, Mhapsekar R, et al (2011) Does the hepatocellular phase of gadobenate dimeglumine help to differentiate hepatocellular carcinoma in cirrhotic patients according to histological grade? Clin Radiol 66: 845–852. 10.1016/j.crad.2011.03.021 21771548

[32] Panic N, Leoncini E, de Belvis G, Ricciardi W, Boccia S, et al (2013) Evaluation of the endorsement of the preferred reporting items for systematic reviews and meta-analysis (PRISMA) statement on the quality of published systematic review and meta-analyses. PLoS One 8 10.1371/journal.pone.0082806 24386151 PMC3873291

[33] Whiting PF, Rutjes AW, Westwood ME, Mallett S, Deeks JJ, et al (2011) QUADAS-2: a revised tool for the quality assessment of diagnostic accuracy studies. Ann Intern Med 155: 529–536. 10.7326/0003-4819-155-8-201110180-00009 22007046

[34] Higgins JP, Thompson SG, Deeks JJ, Altman DG (2003) Measuring inconsistency in meta-analyses. Bmj 327: 557–560. 12958120 10.1136/bmj.327.7414.557 PMC192859

[35] Vamvakas EC. (1998) Meta-analyses of studies of the diagnostic accuracy of laboratory tests: a review of the concepts and methods. Arch Pathol Lab Med 122: 675–686. 9701328

[36] Honest H, Khan KS (2002) Reporting of measures of accuracy in systematic reviews of diagnostic literature. BMC Health Serv Res 2: 7 11884248 10.1186/1472-6963-2-4 PMC100326

[37] Arends LR, Hamza TH, van Houwelingen JC, Heijenbrok-Kal MH, Hunink MG, et al (2008) Bivariate random effects meta-analysis of ROC curves. Med Decis Making 28: 621–638. 10.1177/0272989X08319957 18591542

[38] Zamora J, Abraira V, Muriel A, Khan K, Coomarasamy A (2006) Meta-DiSc: a software for meta-analysis of test accuracy data. BMC Med Res Methodol 6: 31 16836745 10.1186/1471-2288-6-31 PMC1552081

[39] Song F, Khan KS, Dinnes J, Sutton AJ (2002) Asymmetric funnel plots and publication bias in meta-analyses of diagnostic accuracy. Int J Epidemiol 31: 88–95. 11914301 10.1093/ije/31.1.88

[40] EDMONDSON HA, STEINER PE (1954) Primary carcinoma of the liver: a study of 100 cases among 48,900 necropsies. Cancer 7: 462–503. 13160935 10.1002/1097-0142(195405)7:3<462::aid-cncr2820070308>3.0.co;2-e

[41] Enomoto S, Tamai H, Shingaki N, Mori Y, Moribata K, et al (2011) Assessment of hepatocellular carcinomas using conventional magnetic resonance imaging correlated with histological differentiation and a serum marker of poor prognosis. Hepatol Int 5: 730–737. 10.1007/s12072-010-9245-8 21484138 PMC3090556

[42] Kim AY, Kim YK, Lee MW, Park MJ, Hwang J, et al (2012) Detection of hepatocellular carcinoma in gadoxetic acid-enhanced MRI and diffusion-weighted MRI with respect to the severity of liver cirrhosis. Acta Radiol 53: 830–838. 10.1258/ar.2012.120099 22847903

[43] Kim HY, Choi JY, Kim CW, Bae SH, Yoon SK, et al (2012) Gadolinium ethoxybenzyl diethylenetriamine pentaacetic acid-enhanced magnetic resonance imaging predicts the histological grade of hepatocellular carcinoma only in patients with Child-Pugh class A cirrhosis. Liver Transpl 18: 850–857. 10.1002/lt.23426 22407909

[44] Padhani AR, Liu G, Koh DM, Chenevert TL, Thoeny HC, et al (2009) Diffusion-weighted magnetic resonance imaging as a cancer biomarker: consensus and recommendations. Neoplasia 11: 102–125. 19186405 10.1593/neo.81328 PMC2631136

[45] Tamura S, Kato T, Berho M, Misiakos EP, O'Brien C, et al (2001) Impact of histological grade of hepatocellular carcinoma on the outcome of liver transplantation. Arch Surg 136: 25–30. 11146770

[46] Kim I, Kim MJ (2012) Histologic characteristics of hepatocellular carcinomas showing atypical enhancement patterns on 4-phase MDCT examination. Korean J Radiol 13: 586–593. 10.3348/kjr.2012.13.5.586 22977326 PMC3435856

[47] Yu MH, Kim JH, Yoon JH, Kim HC, Chung JW, et al (2014) Small (</ = 1-cm) hepatocellular carcinoma: diagnostic performance and imaging features at gadoxetic acid-enhanced MR imaging. Radiology 271: 748–760. 10.1148/radiol.14131996 24588677

[48] Erturk SM, Ichikawa T, Sano K, Motosugi U, Sou H, et al (2008) Diffusion-weighted magnetic resonance imaging for characterization of focal liver masses: impact of parallel imaging (SENSE) and b value. J Comput Assist Tomogr 32: 865–871. 10.1097/RCT.0b013e3181591cf2 19204445

[49] Goshima S, Kanematsu M, Kondo H, Yokoyama R, Kajita K, et al (2008) Diffusion-weighted imaging of the liver: optimizing b value for the detection and characterization of benign and malignant hepatic lesions. J Magn Reson Imaging 28: 691–697. 10.1002/jmri.21467 18777553

[50] Girometti R, Del Pin M, Pullini S, Cereser L, Como G, et al (2013) Accuracy of visual analysis vs. apparent diffusion coefficient quantification in differentiating solid benign and malignant focal liver lesions with diffusion-weighted imaging. Radiol Med 118: 343–355. 10.1007/s11547-012-0873-z 22986693

[51] Lim KS (2014) Diffusion-weighted MRI of hepatocellular carcinoma in cirrhosis. Clin Radiol 69: 1–10. 10.1016/j.crad.2013.07.022 24034549

[52] Yamada I, Aung W, Himeno Y, Nakagawa T, Shibuya H (1999) Diffusion coefficients in abdominal organs and hepatic lesions: evaluation with intravoxel incoherent motion echo-planar MR imaging. Radiology 210: 617–623. 10207458 10.1148/radiology.210.3.r99fe17617

[53] Kakite S, Dvvorne H, Besa C, Cooper N, Facciuto M, et al (2014) Hepatocellular carcinoma: Short-term reproducibility of apparent diffusion coefficient and intravoxel incoherent motion parameters at 3.0T. LID - J Magn Reson Imaging 10: 24538 10.1002/jmri.24538. 24415565

[54] Taouli B, Koh DM. (2010) Diffusion-weighted MR imaging of the liver. Radiology 254: 47–66. 10.1148/radiol.09090021 20032142

[55] Kim HY, Choi JY, Kim CW, Bae SH, Yoon SK, et al (2012) Gadolinium ethoxybenzyl diethylenetriamine pentaacetic acid-enhanced magnetic resonance imaging predicts the histological grade of hepatocellular carcinoma only in patients with Child-Pugh class A cirrhosis. Liver Transpl 18: 850–857. 10.1002/lt.23426 22407909

[56] Lee MH, Kim SH, Park MJ, Park CK, Rhim H (2011) Gadoxetic acid-enhanced hepatobiliary phase MRI and high-b-value diffusion-weighted imaging to distinguish well-differentiated hepatocellular carcinomas from benign nodules in patients with chronic liver disease. AJR Am J Roentgenol 197 10.2214/AJR.10.6198 22021534

[57] Nasu K, Kuroki Y, Sekiquchi R, Kazama T, Nakajima H (2006) Measurement of the apparent diffusion coefficient in the liver: is it a reliable index for hepatic disease diagnosis? Radiat Med 24: 438–444. 16958425 10.1007/s11604-006-0053-y

[58] Schmid-Tannwald C, Jiang Y, Dahi F, Rist C, Sethi I, et al (2013) Diffusion-weighted MR imaging of focal liver lesions in the left and right lobes: is there a difference in ADC values? Acad Radiol 20: 440–445. 10.1016/j.acra.2012.10.012 23498984

[59] Witjes CD, Willemssen FE, Verheij, van der Veer SJ, Hansen BE, et al (2012) Histological differentiation grade and microvascular invasion of hepatocellular carcinoma predicted by dynamic contrast-enhanced MRI. J Magn Reson Imaging 36: 641–647. 10.1002/jmri.23681 22532493

[60] Saito K, Morivasu F, Sugimoto K, Nishio R, Saguchi T, et al (2011) Diagnostic efficacy of gadoxetic acid-enhanced MRI for hepatocellular carcinoma and dysplastic nodule. World J Gastroenterol 17: 3503–3509. 10.3748/wjg.v17.i30.3503 21941417 PMC3163248

[61] Pirisi M, Leutner M, Pinato DJ, Avellini C, Carsana L, et al (2010) Reliability and reproducibility of the edmondson grading of hepatocellular carcinoma using paired core biopsy and surgical resection specimens. Arch Pathol Lab Med 134: 1818–1822. 10.1043/2009-0551-OAR1.1 21128781

[62] Pawlik TM, Gleisner AL, Anders RA, Assumpcao L, Maley W, et al (2007) Preoperative assessment of hepatocellular carcinoma tumor grade using needle biopsy: implications for transplant eligibility. Ann Surg 245: 435–442 17435551 10.1097/01.sla.0000250420.73854.ad PMC1877015

